# Managing burn patients in a fire disaster: Experience from a burn unit in Bangladesh

**DOI:** 10.4103/0970-0358.70733

**Published:** 2010-09

**Authors:** S. R. Mashreky, S. Bari, S. L. Sen, A. Rahman, T. F. Khan, F. Rahman

**Affiliations:** Centre for Injury Prevention and Research Bangladesh (CIPRB), Bangladesh; 1 Burn Unit, Dhaka Medical Colleges, Bangladesh; 2 Centre for Medical Education, Bangladesh

**Keywords:** Disaster management, fire disaster, problems in inferno

## Abstract

Although burn disaster is not a frequent event, with urbanisation and industrialisation, burn disaster is becoming an emerging problem in Bangladesh. On 3 June 2010, a fire disaster killed 124 people in Neemtali, Dhaka, Bangladesh. This paper narrates the management of burn patients of this disaster in the burn unit of Dhaka Medical College Hospital. The burn unit managed 192 burn victims of the disaster. Forty-two victims were admitted and 150 of them received primary care at the emergency room and were sent back home. Ten patients among 42 in-patients died. The in-patient mortality was 23.8%. Burn unit in Dhaka Medical College Hospital is the only burn management centre in Bangladesh. Proper planning and coordinated effort by all sectors and persons concerned were the key elements in this successful management.

## INTRODUCTION

Fire disaster is a major public health problem in Bangladesh.[1,2] Every year, more than 250,000 people get injured and more than 3000 die due to burn in the country.[3] The worst fire related disaster occurred on 3 June 2010, killing 124 people.

Natural or man-made disasters are the challenges for the world. It becomes more challenging when resources and technology are inadequate. Management of such an event with limited resources in a low income country like Bangladesh requires documentation and sharing of the experiences for future reference. This could be useful in similar situations in other countries with similar socioeconomic and environmental conditions. This paper narrates the process of management of burn victims in one of the major disasters in Dhaka, Bangladesh.

## LOCATION OF THE FIRE DISASTER

**Figure 1:**
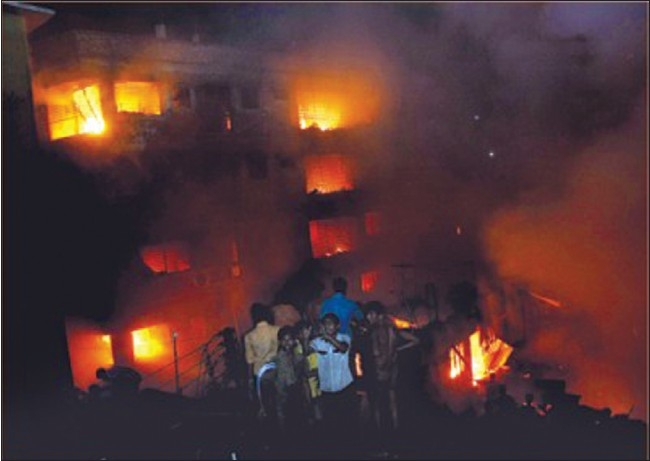
Flames sweep through all floors of the building in the worst fire disaster the country has ever seen

Neeemtali is part of old Dhaka city and is one of the usual old dwellings in third world countries. It is overcrowded with poorly designed buildings and very narrow roads. It is devoid of fire fighting facilities and emergency exit. Many small industries have sprung up, producing combustible chemicals. The whole scenario of the location is fit for major disaster and it is unavoidable to prevent massive death tolls in such a situation [Figure 1].

The kitchen of a wedding ceremony was the source of the fire. The fire spread to the chemicals kept in the ground floor of the same building, which exploded later. Fire engulfed 14 houses including five multistoried residential buildings and five small-scale industrial units. It took about 3 hours to put off the fire. Rescue operation continued for long. The bodies of dead and injured were removed and were laid out on a long stretch of the old Dhaka road. Hundreds of people were injured with differing severity.

## IN BURN UNIT, DHAKA MEDICAL COLLEGE HOSPITAL

**Figure 2:**
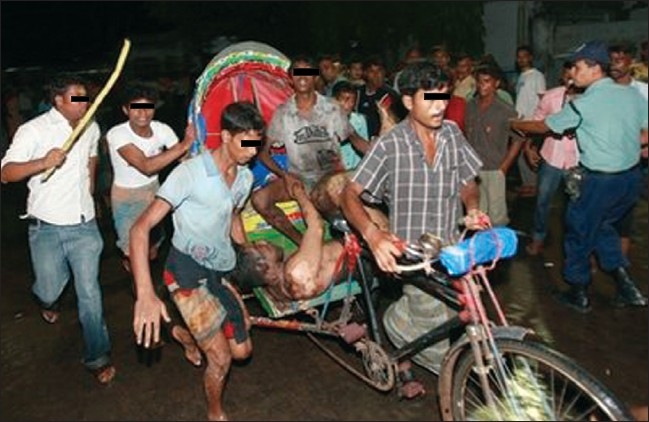
Local rescuers transport a person injured in a fire for treatment to the Burn Unit

At around 21:30 hours, patients started arriving to the burn unit. The Director of the medical college was informed. Simultaneously, the burn unit staffs were informed to get prepared to receive the mass casuality. All of them immediately arrived to the burn unit [Figure 2].

The burn cases were brought to the unit in various available modes of transport. Within a short period of time, the hospital compound, all the rooms and corridors were filled in with burn victims and their accompanying relatives and rescuers. (All the attendants were trying to cool their patients with water using the hospital supply flooding the floor of the burn unit.)

One hundred and ninety-two burn patients attended the emergency department. Forty-two of them were admitted in the burn centre and the other 150 were sent back home with initial management. They were advised to come for follow-up. Thirteen people were brought dead. People were visiting the emergency department in search of their near and dear. It was a heart rendering and pathetic scene filled with painful moaning and crying of the victims and relatives.

## FIRST RESPONSE OF THE HOSPITAL ADMINISTRATORS

The directors of Burn Unit and Dhaka Medical College Hospital together arranged extra manpower to tide over the emergency situation. Fifteen doctors from different units and 70 nursing students were asked to join the emergency management team. Within 30 minutes, the management team was ready with a good number of medical and paramedical personnel. The team was divided into two groups, one team was in the emergency department for giving first aid and other was deputed in the in-patient department for receiving and management. Both the directors were supervising the team serving in the emergency department. The professor of the burn unit was the team leader for in-patients management group.

### Crowd management

**Figure 3:**
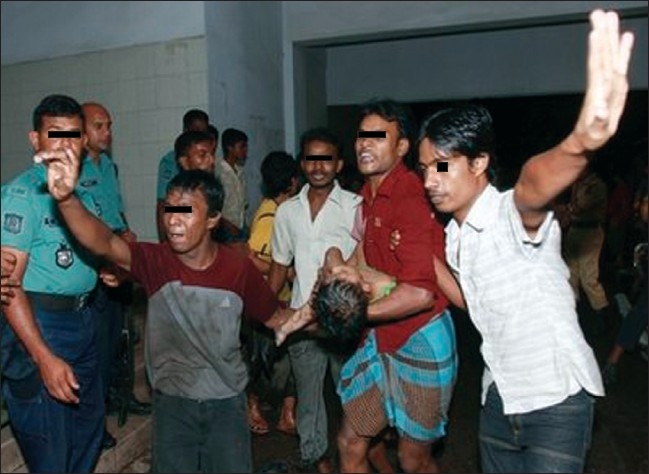
Local rescuers carry a young victim of a fire, one of the gates of Burn Unit

Crowd management in the emergency room was one of the most difficult parts of the disaster management. A large number of patients came to the emergency room in a very short time with their relatives and attendants. It became difficult for the attending doctors and nurses to provide care to the burn victims. Meanwhile, the Minister of Health and Family Welfare arrived at the hospital. With his initiative, the police and other law enforcement departments took charge of crowd control. First 13 dead bodies were sent to the morgue. However, the crowd management was not very satisfactory on the first day [Figure 3].

### Emergency management

The team in the emergency was dressing the wounds with antimicrobial cream and bandage. Those who needed admission were sent to the in-patient department while the rest were sent home and advised for follow-up visits.

### In the in-patient department

#### Challenges

**Figure 4:**
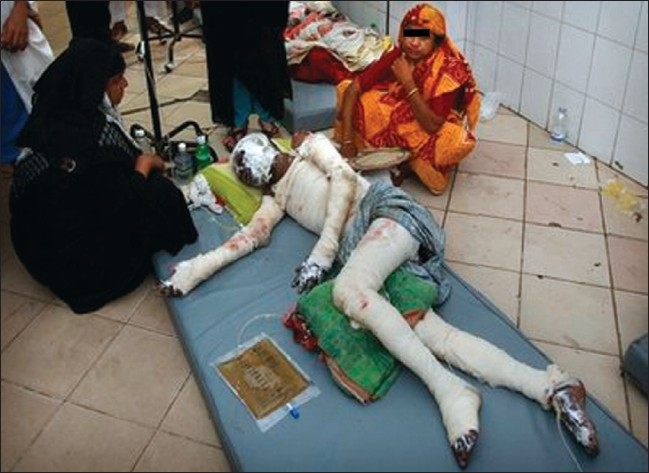
Moslem Uddin, 57, lies on the corridor of Burn Unit of Medical College Hospital

The biggest challenge was managing the crowd within the limited space in the corridor of the burn unit. Overcrowding was a major barrier in delivering the medical treatment. Another challenge was to accommodate the admitted patients in the in-patient department. The 50-bedded burn unit was already treating 250 patients on the day of disaster [Figure 4].

Arranging resources also became a challenge. There were not enough stands for hanging intravenous fluid bags and oxygen cylinders to provide support to those who needed the most.

#### Solution

Corridors were chosen as space for placing the patients on mattresses. Bandages were hung across the corridor and ward to hang the intravenous fluid bags. Fifteen oxygen cylinders were borrowed from Dhaka Medical College Hospital. The availability of drugs and medicines was ensured by the authority.

### Secondary survey and pain management

After initial treatment of all the patients, a team of consultants performed secondary survey and pain management. During this phase, the professor and the consultant physician advised catheterisation and ryle’s tube insertion, where needed. Two patients with inhalational injury and one with 90% total burn surface area (TBSA) were shifted to Intensive Care Unit (ICU) for better management. Two patients had associated head injury, pelvic and femur fractures. The respective consultants were called in for proper management. The patients were given parenteral analgesics to ease their pain.

After secondary survey, first phase was accomplished. On final count, about 150 patients were managed in emergency over a period of 2½ hours and 42 patients were admitted with different severities of burn. There were 23 males and 19 females with age range of 2–57 years, including 10 children below the age of 12 years. Thirty-one patients suffered from ≤40% TBSA burn. Six of them had inhalational injury.

### Support from specially trained personnel

In the burn management team eight persons were specially trained in Emergency Management of Severe Burn (EMSB) which helped in smoother performance in burn management during the disaster.

### Support from Bangladesh army and navy

A medical team from Bangladesh Army and another from Bangladesh Navy were deployed to the burn unit as additional help. They started sharing the responsibility within 12 hours of the incidence.

### Support from highest level of the state

Support was ensured from the highest level of the state. The Health Minister stayed in the burn unit and personally supervised the emergency situation. His support to involve law enforcement authority and to ensure the supply of medicine was of great help. Other ministers, members of parliament and government officials also visited the unit and facilitated the management of patients. Later, the President, the Prime Minister and the leader of the opposition also visited the burn unit, which was encouraging to the team. The Food and Disaster Ministry ensured availability of food for the burn victims, and the government extended financial help including compensation.

### On day two

On the second day, as per the instruction of the Prime Minister, 14 sick patients were referred to the combined military hospital for intensive care. Eleven of them had burn >40 TBA and five had inhalation injury. This created space for rest of the victims. As the emergency crisis was over, the burn cases started getting better coordinated medical service on a regular basis.

### Outcome

Ten of the 42 in-patients died in the hospital. The mortality was 23.8%. Four of them died within 24 hours of the admission. On the third day, another patient with head injury succumbed in the neurosurgery. Four patients with inhalation injury died between 4 and 10 days after admission in the Combined Military Hospital. Rest of the 32 patients survived but some of them would require reconstructive surgery.

## DISCUSSION

Casualty in a fire disaster depends on circumstances, cause and place of disaster. In a residential place where the approach for fire service or other rescuer is difficult, a fire accident will cause higher casualties. In Neemtali, flammable chemicals stored in a residential building caused the disaster. The fire fighting service was delayed as the site was unapproachable because of narrow roads. Hence, the planning of disaster management should start from improving civic amenities. Public awareness and designing and enforcement of special law could go a long way in preventing such a disaster.

Management of patients after a disaster can play an important role in reducing casualties. In fire disasters, hospital mortality was 6% in Netherlands and 54% in Ghana.[4,5] In the disaster in Bangladesh reported here, the mortality rate was 24%. This mortality rate depended upon many of the issues like, extent and nature of burn, available facilities and resources for prompt burn management.

Management of a disaster has phases of planning, preparedness, training, response, relief, rehabilitation and reconstruction.[6] The Burn Unit of Dhaka Medical College Hospital managed the burn disaster efficiently with its efficient leadership and support from the highest level of the state. However, there are no established documents on any structured plan for managing such burn disasters in Bangladesh.

The management of fire disaster includes three phases: pre-hospital response, first-level hospital response and second-level hospital response.[7] It was observed here that the majority of the patients arrived in the burn unit without any triage or pre-hospital care. Triage was done in the emergency department of the centre. Both the first and second levels of hospital care were provided in the burn unit as the incident happened very near to this centre.

Triage is very important in systematic management of a mass casualty. Burn casualty triage is conditioned by the number of patients, the gravity of the burns, the age of the patients, the presence of respiratory complications, and the availability of beds. It is useful to decide the management protocol for a patient based on the gravity of situation.[8]

A team of 10 doctors and 32 paramedical staff under direct supervision of the head of the institute at first contact of service delivery in emergency was a major strength of the disaster management plan. This team did triage, provided first aid and took decision regarding the management of each and every patient. The large number of trained manpower in in-patient department, including a professor and two consultants, made the management smooth. The administrative help in arranging the required equipments, oxygen, IV fluids, drugs and dressing material ensured a successful implementation of the orders. Certain innovative methods are needed to adopt in a limited resource. The cooperation and coordination between different medical and nursing institutes in terms of manpower, material and machine are keys in the management of a disaster.

### Major strengths in managing the disaster

- Instant and appropriate planning of the burn centre
- Presence of adequate number of medical and paramedical personnel
- Very good coordination between burn centre and the adjacent medical institute
- Full time monitoring and supervision by the heads of two institutions
- Availability of personnel with specialised training in burn management
- Administrative support by the highest level of the state
